# Comparative Evaluation of Mandibular Ridge Resorption in Post-menopausal Denture and Non-Denture-Wearing Females and Its Relation to Body Mass Index: An In-Vivo Study

**DOI:** 10.7759/cureus.72757

**Published:** 2024-10-31

**Authors:** Mariyam Zaki, Susmita Roy, Rupa Singh, Futoon Abdulaziz Abed, Shomoukh Alsharifghazi Alabdali, Mohammed Shammas

**Affiliations:** 1 Prosthodontics, Career Post Graduate Institute of Dental Sciences and Hospital, Lucknow, IND; 2 Prosthodontics and Crown and Bridge, Dent-Arch Multispeciality Dental Clinic and Implant Centre, Agartala, IND; 3 Prosthodontics and Crown and Bridge, Mithila Minority Dental College and Hospital, Darbhanga, IND; 4 General Dentistry, Roots Dental Clinic, Jeddah, SAU; 5 General Dentistry, Alnahdi Care Clinic, Jeddah, SAU; 6 Oral and Maxillofacial Rehabilitation, Ibn Sina National College, Jeddah, SAU

**Keywords:** body mass index, denture-wearing, mandibular ridge resorption, post-menopausal women, prosthodontics

## Abstract

Background

Mandibular ridge resorption is a common problem in edentulous individuals, particularly in post-menopausal women. Body mass index (BMI) has been suggested as a potential factor influencing ridge resorption, but the relationship between the two remains unclear.

Methods

A study was conducted to evaluate mandibular ridge resorption in post-menopausal denture-wearing females and its relation to body mass index. A sample of 60 completely edentulous post-menopausal women was divided into two groups based on denture-wearing status and BMI. A radiographic evaluation of mandibular ridge resorption was conducted, and statistical analysis was performed using correlation and regression analysis.

Results

The study found a statistically significant positive correlation between BMI and mandibular ridge resorption in denture-wearing subjects, with a p-value of 0.039. A slight negative correlation was observed in non-denture wearers, with a p-value of 0.122. Overall, a significant relationship was found between BMI and mandibular ridge resorption, with a p-value of 0.007.

Conclusion

The study highlights the importance of considering BMI as a factor in mandibular ridge resorption in post-menopausal denture-wearing females. The findings suggest that prosthodontists should aim to preserve the residual ridge by using techniques such as broad area coverage, reduced denture teeth, and optimal occlusal tooth design.

## Introduction

The typical function of bones is to undergo continual remodeling throughout life by the process of bone resorption and bone creation. Furthermore, both are typically in equilibrium except during growth, when bone synthesis exceeds bone resorption. As aging advances, this equilibrium is lost, and consequently, bone resorption outweighs bone creation [1]. Residual ridge resorption (RRR) is a chronic, persistent, and cumulative multifactorial process of bone remodeling. Additionally, bone loss is larger during the first few months after tooth extraction than later, since it slows down with time after extraction [2]. Many systemic and local variables are connected with residual ridge resorption. Some of these factors include nutritional deficiencies, hormonal imbalance, metabolic bone disorders, hormonal or medication intake, age, and gender [2].

The severity of bone loss and high incidence of RRR are usually connected with osteoporosis. This connection increases with the advancement of age and favors the female sex for predisposition [2]. Local factors associated with RRR include the quality, size, and shape of a residual ridge after tooth extraction, denture-wearing habits, loading of alveolar ridges following tooth loss, quality of dentures, stability of dentures, duration of edentulousness, incorrect horizontal and vertical jaw relation, nighttime wearing of dentures, and reduced masticatory forces in denture wearers [2]. Numerous investigators have attempted to examine the changes in the form of residual ridges using lateral cephalograms, denture relining methods, and diagnostic castings [3].

The resorption of the jaw bones can be assessed by calculating the vertical height of the remaining ridge using panoramic radiography. Panoramic radiography is a widely used method of screening patients and is one of the most commonly employed diagnostic modalities [4]. Therefore, this in-vivo investigation was carried out to investigate ridge resorption in post-menopausal females and its association with body mass index.

## Materials and methods

Study design and setting

This prospective, in-vivo study was conducted in the Department of Prosthodontics and Crown & Bridge and Department of Oral Medicine, Diagnosis, and Radiology of Career Post Graduate Institute of Dental Sciences & Hospital, Lucknow, India, over a span of two years, with CPGIDSH/1296/16 being the registration number assigned to this investigation.

Patient selection

The study was limited to completely edentulous post-menopausal women who reported to the department for replacement of their missing teeth or maintenance of their existing complete dentures. The study sample consisted of 60 subjects, which was further divided into two groups: Group A (30 subjects) comprising denture-wearing subjects, and Group B (30 subjects) comprising non-denture-wearing subjects. Additionally, the study sample was divided into two groups based on body mass index (BMI): Group C (subjects with BMI less than 24) and Group D (subjects with BMI equal to or greater than 24).

Inclusion and exclusion criteria

The study's inclusion criteria were limited to completely edentulous female patients who visited the department, patients without any intraoral lesions, and patients free from any systemic diseases. On the other hand, the exclusion criteria consisted of edentulous male patients, patients who were clinically edentulous but had impacted teeth or root stumps on radiographic examination, patients with traumatic injuries, and radiographs where the mental foramen was not easily distinguishable.

Parameters assessed

The height and weight of the subjects were measured using a stature meter and a digital weighing machine, respectively. The body mass index (BMI) of each subject was calculated by dividing the weight (kg) of the subject by the square of her height (m^2^).

Radiographic evaluation

A static panoramic radiograph was made using an orthopantograph machine. The right mental foramen was identified and divided into two halves. The amount of residual ridge resorption was estimated using the formula given by Wical and Swoope: R = 3x-L (where R: amount of mandibular RRR; x: distance from the inferior border of the mandible to the lower border of the mental foramen; L: height of the mandibular residual alveolar ridge).

Statistical analysis

Statistical analysis of the data was conducted using Pearson's correlation, Spearman's rank correlation, and regression analysis. A comparative study was conducted on 60 edentulous patients, who were divided into four groups based on denture-wearing status and BMI. Measurements of height, weight, distance between the mandible and mental foramen, and actual ridge height were recorded using a measurement tool.

## Results

The distribution of subjects according to denture-wearing and BMI groups is shown in Table 1.

**Table 1:** Distribution of subjects according to denture wearing (Group A and Group B) and BMI groups (Group C and D) BMI: body mass index

Distribution of subjects		BMI group				Total
		Group C		Group D		
		No. of subjects	%	No. of subjects	%	
Denture-wearing/non-denture-wearing	Group A	15	50.0	15	50.0	30
	Group B	9	30.0	21	70.0	30
Total		24	40.0	36	60.0	60

Within Group A, 15 subjects (50%) had a BMI less than 24 (Group C), and 15 subjects (50%) had a BMI equal to or more than 24 (Group D). Similarly, within Group B, nine subjects (30%) had a BMI less than 24 (Group C), and 21 subjects (70%) had a BMI equal to or more than 24 (Group D). The actual BMI values of the subjects are shown in Table 2.

**Table 2:** Distribution of subjects according to denture wearing (Group A and Group B) and actual BMI BMI (kg/m²): body mass index; SD: standard deviation

Denture-wearing/non-denture-wearing	No. of subjects	BMI			
		Mean	SD	Minimum	Maximum
Group A	30	24.18	4.43	16.58	33.37
Group B	30	25.87	3.44	18.57	36.42
Total	60	25.03	4.02	16.58	36.42

The results showed that the mean BMI of the denture wearers (Group A) was 24.18, with a standard deviation of 4.43, ranging from a minimum of 16.58 to a maximum of 33.37. The mean BMI of the non-denture wearers (Group B) was 25.87, with a standard deviation of 3.44, ranging from a minimum of 18.57 to a maximum of 36.42. Overall, the total mean BMI of all 60 subjects was 25.03, with a standard deviation of 4.02, ranging from a minimum of 16.58 to a maximum of 36.42. The correlation between BMI and RRR among denture cases was analyzed (Table 3).

**Table 3:** Correlation between BMI and RRR among denture cases (*p < 0.05, significant correlation) BMI (kg/m²): body mass index; RRR (cm): residual ridge resorption

Group	Factor	BMI	RRR (cm)	Pearson correlation	p-value	Spearman rank correlation	p-value
Both	BMI	1	-0.345	-	0.007	-0.368	0.004
Both	RRR	-0.345	1	0.007	-	-0.368	0.004
A	BMI	1	-0.379	-	0.039	-0.359	0.049
A	RRR	-0.379	1	0.039	-	-0.359	0.049
B	BMI	1	-0.288	-	0.122	-0.234	0.212
B	RRR	-0.288	1	0.122	-	-0.234	0.212

In the combined group of denture-wearing and non-denture-wearing subjects, a negative correlation of -0.345 was found between BMI and RRR, with a p-value of 0.007, indicating a statistically significant relationship. Additionally, a Spearman's rank correlation of -0.368 was observed, with a p-value of 0.004, further supporting the significant association between BMI and RRR. In the group of denture-wearing subjects (Group A), a negative correlation of -0.379 was found between BMI and RRR, with a p-value of 0.039, indicating a statistically significant relationship. Similarly, a Spearman's rank correlation of -0.359 was observed, with a p-value of 0.049, supporting the significant association between BMI and RRR in this group. In contrast, in the group of non-denture-wearing subjects (Group B), a negative correlation of -0.288 was found between BMI and RRR, with a p-value of 0.122, indicating a non-significant relationship. A Spearman's rank correlation of -0.234 was also observed, with a p-value of 0.212, further supporting the non-significant association between BMI and RRR in this group.

As shown in Table 4, the mean RRR was significantly higher in Group C (BMI < 24) compared to Group D (BMI ≥ 24), with a mean difference of 0.242 cm (p = 0.036).

**Table 4:** Comparison of mean RRR between BMI groups and denture groups (*p < 0.05, significant difference) BMI (kg/m²): body mass index; RRR (cm): residual ridge resorption; SD: standard deviation

Group	No. of subjects	Mean RRR (cm)	SD	t-value	p-value
Group C (BMI < 24)	24	1.175	0.437	2.149	0.036
Group D (BMI ≥ 24)	36	0.933	0.420	-	-
Group A (denture-wearing) - Group C	15	1.227	0.454	1.797	0.083
Group A (denture-wearing) - Group D	15	0.960	0.352	-	-
Group B (non-denture-wearing) - Group C	9	1.089	0.417	0.962	0.344
Group B (non-denture-wearing) - Group D	21	0.914	0.470	-	-
Group A (denture-wearing)	30	1.093	0.422	1.118	0.268
Group B (non-denture-wearing)	30	0.967	0.455	-	-
Group A (denture-wearing) - Group C	15	1.227	0.454	0.741	0.467
Group B (non-denture-wearing) - Group C	9	1.089	0.417	-	-
Group A (denture-wearing) - Group D	15	0.960	0.352	0.318	0.753
Group B (non-denture-wearing) - Group D	21	0.914	0.470	-	-

Among the denture-wearing subjects (Group A), the mean RRR was higher in Group C compared to Group D, but the difference was not statistically significant (p = 0.083). Similarly, among the non-denture-wearing subjects (Group B), the mean RRR was higher in Group C compared to Group D, but the difference was not statistically significant (p = 0.344). When comparing the mean RRR between the two denture groups (Group A and Group B), the results showed that the mean RRR was higher in Group A compared to Group B, but the difference was not statistically significant (p = 0.268). Subgroup analyses were also conducted to compare the mean RRR between the two denture groups within each BMI group. Among subjects with BMI < 24 (Group C), the mean RRR was higher in Group A compared to Group B, but the difference was not statistically significant (p = 0.467). Similarly, among subjects with BMI ≥ 24 (Group D), the mean RRR was higher in Group A compared to Group B, but the difference was not statistically significant (p = 0.753).

The regression analysis (Table 5) showed that there was a significant negative relationship between RRR and BMI in both groups, with a regression coefficient of -0.038 (p < 0.05).

**Table 5:** Regression analysis for RRR and BMI (*p < 0.05, significant regression coefficient) BMI (kg/m²): body mass index; RRR (cm): residual ridge resorption; SE: standard error

Group	Dependent variable	Independent variable	Regression coefficient	SE	R2
Both (A and B)	RRR (y)	BMI	-0.038	0.013	0.119
		Constant	1.975	0.341	
Group A (denture-wearing)	RRR (y)	BMI	-0.050	0.023	0.144
		Constant	2.264	0.603	
Group B (non-denture-wearing)	RRR (y)	BMI	-0.027	0.017	0.083
		Constant	1.758	0.424	

This suggests that for every unit increase in BMI, RRR decreased by 0.038 units. In the denture-wearing group (Group A), the regression analysis revealed a stronger negative relationship between RRR and BMI, with a regression coefficient of -0.050 (p < 0.05). This indicates that for every unit increase in BMI, RRR decreased by 0.050 units in this group. In the non-denture-wearing group (Group B), the regression analysis showed a weaker negative relationship between RRR and BMI, with a regression coefficient of -0.027 (p < 0.05). This suggests that for every unit increase in BMI, RRR decreased by 0.027 units in this group.

## Discussion

At present, a growing number of edentulous individuals are managing their lives despite numerous serious health issues. Edentulism has been demonstrated to have a highly marked effect on the rate of residual ridge resorption, which contributes to an augmentation in alveolar bone height increase and the area supporting a denture. The lack of natural teeth compromises a patient's masticatory efficiency because nature designed humans to chew with their teeth. In addition, complete denture users require nearly seven times more chewing movements than those with natural teeth to chew food to half its original size. The absence of teeth can also impact speech articulation in partially edentulous patients [5].

RRR is a chronic, progressive, irreversible process with multiple contributing factors [6]. Such factors are usually subdivided into four categories: anatomic, metabolic, functional, and prosthetic. Anatomic factors include ridge size and shape, bone type, and mucoperiosteum characteristics. Metabolic factors encompass such aspects as age, sex, hormonal balance, and conditions like osteoporosis. Functional factors involve the frequency, direction, and magnitude of the forces applied to the ridge [7]. A known fact is that estrogen plays a primary role in the development of bone, maturation of the bone, and the overall regulation of bone turnover. In bone formation, it is established that estrogen provides the necessary function of closing the epiphyseal growth plates in both males and females [8]. The postmenopausal reduction in estrogen causes a decrease in calcium absorption and utilization, which in turn triggers osteoporosis, a key factor in bone loss [9]. Other investigators have found that the period of edentulism is the most significant variable for the amount of mandibular bone resorption [10].

Residual ridge atrophy reduces the support that dentures provide, which makes it a persistent issue in prosthodontics. Researchers are also still debating whether denture use causes atrophy of the ridge. Jozefowicz found that residual ridges of denture-wearing patients were significantly smaller than those of non-denture wearers [11]. Pietrokovski et al. reported on a case whereby the residual ridges of the non-denture wearer were significantly wider. Canger et al. reported that vertical ridge heights were greater in non-denture wearers than in denture users, and the differences were greater in the mandible, whereas the maxillary ridge heights did not differ between the two populations [12].

BMI has also been shown to be a related factor for atrophy of the jawbone; however, not all patients have atrophy of the jawbone along with lower bone mineral density and vice versa. Kroger et al. suggested that a low-height, light-bodied nulliparous woman is at greater risk for more severe osteoporosis than a taller, heavier woman who has given birth. The reason varies with differences in both bone mass and estrogen metabolism, that is, related to levels of body fat [13]. Klemetti et al. have reported that the residual mandibular ridges were markedly higher in subjects with a higher BMI than with a lower BMI [14]. Several methodologies have been developed to assess RRR, including cephalometric radiographs, dento-contourographs, cast comparators, photogrammetry, and the most commonly used orthopantomogram method. In this study, panoramic radiographs were used to evaluate bone resorption. There are also studies that proved the merit and accuracy of panoramic radiographs, providing reliable data for both linear and angular mandibular measurements [15-18].

In the present study, the mental foramen was considered a reference landmark for assessing ridge resorption. It is one of the reliable and reproducible landmarks used for mandible studies. One of the most applied parameters for clinical investigations was the estimated ratio of total bone height to the height of the foramen above the inferior border regarding the location of the mental foramen in relation to both inferior and superior borders of the mandible [15]. To retain the integrity of the residual ridges, the following prosthodontic steps have to be followed to facilitate their management: maximize the area coverage of the denture base; reduce the number of denture teeth; buccolingual reduction of the width of the tooth; optimal design of the occlusal tooth with respect to the chewing group; centralize the occlusal contacts. An appropriate vertical rest dimension should be established by proper recording of the maxillomandibular relationship, which reduces the frequency and duration of contact between them and thereby provides some rest to the underlying ridges [16].

An appropriate intake of calcium and phosphorus in balanced proportion, with the concurrent administration of vitamin D3, also reduces the activity of bone resorption. Kribbs' experiment illustrated that 1000 mg of calcium and 400 IU of vitamin D3 per day, over two years, prevented mandible bone loss in the affected women with post-menopause conditions, and in certain women, it was observed that their bone mass was even increased. The National Institutes of Health recommends postmenopausal women take between 1000 and 1500 mg daily [17]. Diet in edentulous persons should also provide adequate amounts of fiber from foods, fruits, and vegetables with minimal cholesterol and saturated fat intake [18]. The results of this study showed a raised need for calcium in edentulous patients, and hence interventions such as dietary modifications, hormone replacement therapy, and encouraging them to complete denture fabrication would be helpful.

One of the limitations of this study is that it is based on a small sample size and further divided into several groups. With an overall sample of 60 subjects, statistical power may be particularly compromised where subgroup comparisons have seen the numbers reduced, for example, to just nine in Group B at BMI <24. This may have been one reason that the differences in some of these comparisons did not reach statistical significance, despite trends appearing within the data. Additionally, a cross-sectional study cannot be used to demonstrate causation regarding associations between BMI and RRR. The study also did not control for confounding factors that might have been influencing both the BMI and RRR simultaneously; for example, nutritional status, duration of being edentulous, or other general health conditions. Utilization of BMI alone might also ignore other factors, like fat distribution or muscle mass, which could influence bone resorption processes and thus be relevant in the assessment.

## Conclusions

A significant inverse correlation was observed between BMI and RRR in the overall target population in our study, suggesting that as BMI decreases, RRR increases. Notably, this inverse correlation is also observed in denture-wearing subjects, where a decrease in BMI is associated with an increase in RRR. In contrast, a non-significant negative correlation is observed between BMI and RRR in non-denture-wearing subjects, indicating a lack of statistically significant association between these variables in this subgroup.

## References

[1] AlSheikh HA, AlZain S, Warsy A, AlMukaynizi F, AlThomali A (2019). Mandibular residual ridge height in relation to age, gender and duration of edentulism in a Saudi population: a clinical and radiographic study. Saudi Dent J.

[2] Disha V, Čelebić A, Peršić S, Papić M, Rener-Sitar K (2024). Correction to: orofacial esthetics, chewing function, and oral health-related quality of life in Kennedy class I patients with mini-implant-retained removable partial dentures: a 3-year clinical prospective study. Clin Oral Investig.

[3] Jagadeesh MS, Patil RA, Kattimani PT (2013). Clinical evaluation of mandibular ridge height in relation to aging and length of edentulism. IOSR J Dent Med Sci.

[4] Panchbhai AS (2013). Quantitative estimation of vertical heights of maxillary and mandibular jawbones in elderly dentate and edentulous subjects. Spec Care Dentist.

[5] Emami E, de Souza RF, Kabawat M, Feine JS (2013). The impact of edentulism on oral and general health. Int J Dent.

[6] Atwood DA, Coy WA (1971). Clinical, cephalometric, and densitometric study of reduction of residual ridges. J Prosthet Dent.

[7] Sta Maria MT, Hasegawa Y, Marito P, Yoshimoto T, Salazar S, Hori K, Ono T (2023). The impact of residual ridge morphology on the masticatory performance of complete denture wearers. Heliyon.

[8] Li H, Li G, Yi M (2024). Sex-specific associations of urinary mixed-metal concentrations with femoral bone mineral density among older people: an NHANES (2017-2020) analysis. Front Public Health.

[9] Veldurthy V, Wei R, Oz L, Dhawan P, Jeon YH, Christakos S (2016). Vitamin D, calcium homeostasis and aging. Bone Res.

[10] Ameri N, Alikhasi M, Rezayani V (2017). Full mouth rehabilitation with retrievable metal-ceramic implant-supported fixed prostheses for a young patient with atrophic jaws: a clinical report. Clin Case Rep.

[11] Józefowicz W (1970). The influence of wearing dentures on residual ridges: a comparative study. J Prosthet Dent.

[12] Canger EM, Celenk P (2012). Radiographic evaluation of alveolar ridge heights of dentate and edentulous patients. Gerodontology.

[13] Zlatarić DK, Celebić A, Kobler P (2002). Relationship between body mass index and local quality of mandibular bone structure in elderly individuals. J Gerontol A Biol Sci Med Sci.

[14] Issrani R, Reddy J, Bader AK (2023). Exploring an association between body mass index and oral health-a scoping review. Diagnostics (Basel).

[15] Alshenaiber R, Cowan C, Barclay C, Silikas N (2021). Analysis of residual ridge morphology in a group of edentulous patients seeking NHS dental implant provision-a retrospective observational lateral cephalometric study. Diagnostics (Basel).

[16] CA RL (1960). A comparative study of the resorption of the alveolar ridges in denture-wearers and non-denture-wearers. J Am Dent Assoc.

[17] Kribbs PJ, Chesnut CH 3rd, Ott SM, Kilcoyne RF (1989). Relationships between mandibular and skeletal bone in an osteoporotic population. J Prosthet Dent.

[18] Al-Rafee MA (2020). The epidemiology of edentulism and the associated factors: a literature Review. J Family Med Prim Care.

